# Lessons learned and study results from HIVCore: an HIV implementation science initiative

**DOI:** 10.7448/IAS.19.5.21194

**Published:** 2016-07-20

**Authors:** Naomi Rutenberg, Waimar Tun, Nagesh N Borse

**Affiliations:** 1 HIV and AIDS Program, Population Council, Washington, DC, USA; 2 Research Division, Office of HIV and AIDS, US Agency for International Development (USAID), Washington, DC, USA

The global community has made substantial progress towards halting and beginning to reverse the HIV epidemic. We have seen a 35% decrease in new HIV infections since 2000 and a 42% decrease in AIDS-related deaths since the peak in 2004 [1]. We have effective interventions such as HIV testing and counselling (HTC), prevention of mother-to-child transmission (PMTCT) and voluntary male medical circumcision (VMMC) as part of our tools for curbing the epidemic [2–4]. In addition, scientific results established the effectiveness of antiretrovirals (ARV) to not only treat but also to prevent HIV infections [5–7]. Together these have given us the much needed tools and knowledge to bring the goal of achieving an AIDS-free generation within our reach [8].

Despite great progress, there is a large unfinished agenda in addressing HIV infection and AIDS-related morbidity and mortality [9]. UNAIDS has set forth a goal of 90–90–90 – an ambitious treatment target to help end the AIDS epidemic [10]. By 2020, 90% of all people living with HIV will know their HIV status, 90% of all people with diagnosed HIV infection will receive sustained antiretroviral therapy and 90% of all people receiving antiretroviral therapy will have viral suppression. However, achieving these targets will require unprecedented action to scale up access to the available tools and interventions.

**Figure 1:**
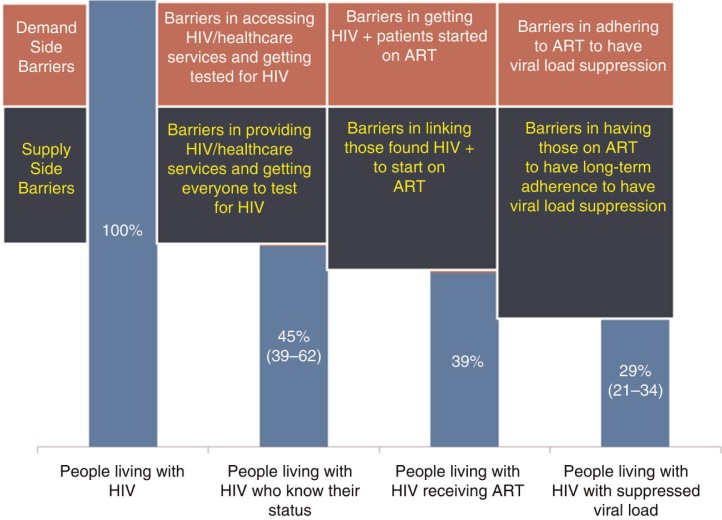
Demand- and supply-side gaps experienced in implementing and accessing HIV treatment among adults in sub-Saharan Africa aged 15 years and more, 2013. Source: UNAIDS 90-90-90 Report, 2014.

As of June 2015, UNAIDS estimated that globally only 43% of people living with HIV were on antiretroviral treatment (ART), leaving nearly 22 million people living with HIV without treatment [1]. The factors that contribute to the large unmet need for HIV treatment include demand and supply barriers as well as stigma and discrimination against people living with HIV and select sub-populations at high risk of HIV [10,11]. Demand-side barriers include gaps in accessing HIV/healthcare services and getting tested for HIV, in timely initiation of ARV treatment by HIV-positive patients and in adherence to ART and achieving viral suppression. On the supply side, there are major gaps in the provision of HIV testing services, in linkages to HIV treatment and in support to patients to initiate and adhere to ART (Figure 1).

In addition to general supply and demand gaps, contextual factors also create barriers. Rural areas tend to have a smaller number of people living with HIV; however, health facilities in rural areas often face severe health systems constraints in human resources, laboratory equipment, and supply of drugs. Although urban areas tend to have better health systems and less infrastructure challenges, they are challenged by a higher demand for HIV services. Compared with the general population, HIV infection rates are substantially higher among men who have sex with men (3–25 times), sex workers (13.5 times) and people who inject drugs (22–50 times) [12–15]. Although the overall prevalence of HIV is falling, epidemics in these key groups are expanding in many places worldwide. Adolescents and people living with disabilities are two more examples of sub-populations that have specific risks of HIV and needs for tailored services [9,13,16]. Prevention and treatment of HIV in these marginalized groups is difficult to address because of stigma, discrimination and their sequelae.

Addressing these barriers requires knowledge about what strategies are effective for the delivery of HIV testing and treatment for the different settings and sub-populations. We know what to do but not always how to do it. The US President's Emergency Plan for AIDS Relief (PEPFAR) implementation science (IS) framework describes IS as the study of methods to improve the uptake, implementation and translation of research findings into routine and common practices [17]. Since 2005, PEPFAR has been working with implementing partners and national governments in many HIV-affected countries and has contributed to the rapid acceleration of HIV treatment access, availability of care and support services and HIV prevention interventions. IS addresses the gaps in the 90–90–90 coverage and is critical for building upon the progress already achieved with PEPFAR funding.

The Population Council and partners from Elizabeth Glaser Pediatric AIDS Foundation, Palladium, and the University of Washington with funding from the US Agency for International Development (USAID), launched HIVCore, which is a five-year operations research/IS initiative, addressing critical service delivery issues in global HIV and AIDS PMTCT and treatment, care and support programmes.

HIVCore addressed three priorities through IS. First, retention in care, that is, meeting current commitments to patients on treatment and promoting prevention by lowering population-level viral loads. Second, the integration of HIV and other health and social services, strengthening linkages between community- and facility-based HIV services and the use of innovations in management information systems and information communication technologies to improve linkages to care. Third, continue to roll out and scale up services at existing service sites, add new sites and expand geographic and population coverage.

This journal supplement provides selected results from the HIVCore initiative. The papers are drawn from seven studies in PEPFAR-supported countries in sub-Saharan Africa. The studies address the demand for and/or delivery of treatment for adults and adolescents, PMTCT programmes, enhancing prevention for marginalized young adults by addressing their mental health, and meeting the HIV-related needs of people living with disabilities. One paper provides information on the costs of different service delivery models. One paper and one commentary address important methodological issues in conducting IS.

A number of studies included in this supplement discovered substantial loss to follow-up (LTFU) or attrition from care of HIV-positive clients, anywhere from one-quarter to three-quarters in the first year after HIV diagnosis. The LTFU is generally most severe in the early months after diagnosis. Woelk *et al*.'s study of PMTCT clients in Rwanda found that unmarried, apparently healthy (i.e. ART ineligible), and women with higher CD4 counts at enrolment were at the greatest risk of LTFU in the sites they studied [18]. In Mozambique, men and women who enrolled early in HIV care said that the main reason for obtaining an HIV test – and for enrolling in HIV care – was the presence of signs or symptoms of sickness [19]. Conversely, the main perceived barrier for enrolment was lack of signs or symptoms of sickness. In Uganda, Okoboi *et al*. describe how retention in care was better among younger adolescents (aged 10–14 years) than older adolescents, adolescents who initiated ART in earlier years of the programme when it had a stronger community focus and adolescents who had higher CD4 counts at ART initiation [20].

Certain vulnerable populations have unique needs for HIV services. Tun *et al*. describe how persons with disabilities in Ghana, Uganda and Zambia face many barriers to accessing services that make it very difficult for them to get tested for HIV and, if found positive, many persons with disabilities are unlikely to initiate treatment and be retained in care and treatment due to a myriad of challenges, including stigma related to both HIV and disabilities, physically inaccessible facilities, lack of accessible information, such as in Braille or sign interpreters, lack of trained staff to provide services for persons with disabilities, economic hardships and those who are illiterate [21].

As noted above, IS is about identifying effective ways to improve implementation and address gaps and challenges. The Jani *et al*. study tested a novel intervention among migrant adolescents in Ethiopia based on a formative assessment [22]. The researchers piloted and evaluated the effects of an intervention to reduce mental health problems and improve HIV-related outcomes among migrant adolescents in Addis Ababa. They found that the psychosocial counselling intervention was associated with increased knowledge and uptake of HIV and sexual health services among both male and female vulnerable adolescents, and reduced mental health problems among female adolescents.

Other studies also yielded signposts to what should be done based on the results of formative evaluations. Granato *et al*. found that sites with better retention in care and treatment tended to have more and better trained staff; better physical infrastructure that offer privacy, confidentiality and a better level of comfort for patients; and these sites actively followed-up HIV-positive patients who missed visits [23]. Activities to promote social support were also associated with higher retention in the Inguane *et al*. study in Mozambique [19]. Other important factors were patients’ perceptions that they would get good care, the financial costs of travel and wait time at clinics. Finally, linkage to care and “re-linkage,” that is re-connecting patients who have dropped out of care, may require different strategies.

A paper by Vu *et al*. provides a description of the cost per ART visit and annual ART-related costs per patient for three different task-shifting models of ART service delivery in Uganda [24]. Unit costs, the distribution of costs and resource utilization varied widely across the three sites and models, suggesting the potential for efficiency gains in ART service delivery. In particular, HIV programmes in Uganda may save costs by reducing the number of annual ART visits to match the national standard (four ART visits a year on average). Further, non-government organizations providing ART services, similar to these three organizations, Kitovu Mobile, The AIDS Support Organisation (TASO), and Uganda Cares may benefit from collaborating with the government and using government facilities to reduce operational costs.

A major challenge for all these studies was the quality of the national data systems. Many of the studies presented in this supplement encountered significant amounts of missing data in the routinely collected national data sets accessed for these studies. They also encountered a significant amount of variation in the number of services reportedly delivered or patients attended, depending on which data source was consulted. In addition, considerable error was introduced as data were aggregated from the clinic to district or provincial to national level. Gloyd *et al*. detail various types of shortcomings in the data, which is a useful checklist for both evaluating the quality of data and considering investments to improve data quality [25]. Researchers would like to think that secondary data analysis will be the “low hanging fruit” and be more efficient and faster given the availability of the data; however, the reality is that the data are often so incomplete and of poor quality that use of these data are highly complex and demanding. While the sheer amount of data that health providers are asked to collect is one source of poor data quality, the silver lining for research is that the volume of data provides the opportunity to compare and triangulate. The authors of the paper describe a variety of approaches, including imputation and sensitivity analysis, for assembling a decent picture of services despite the incomplete data.

The commentary by Kalibala *et al*. highlights the importance of and challenges to paying the same attention to measuring the implementation – that is, the dose, coverage, fidelity and quality – of the interventions as is paid to the outcomes in the target beneficiaries [26]. They remind us that since the purpose of IS is to identify and increase the use of effective service delivery approaches, it is incumbent upon IS studies to evaluate and document these critical elements and publish the processes and materials used in implementation.

Apart from conducting IS to expand the evidence base of effective service delivery approaches at the global, country and programme levels, the HIVCore initiative also focused its efforts on promoting utilization of IS results to enhance decision-making and building local capacity for IS. As an outcome of the engagement of local stakeholders in Ghana in the study on persons with disabilities [21], the Ghana AIDS Commission announced that the new National Strategic Plan (NSP) would be revised to better address the needs of persons with disabilities, as prior to this study Ghana's NSP did not include disability as a challenge in HIV programming, and that disability-accessible educational materials will be developed. Upon completion of the first studies in the initiative, HIVCore convened an international writing workshop to enable sharing of experiences in data analysis and to highlight policy and programmatic implications of study findings. Through the use of experienced investigators and technical advisors, this workshop focused on mentoring less experienced investigators to build their capacity in scientific writing, including writing the first drafts of many of the manuscripts in this supplement.

These IS studies, and others like them, call for attention to the necessary improvements at service delivery points and in sub-national and national systems to expand coverage of HIV care while also strengthening healthcare systems, including data systems, human resources and infrastructure. IS is an iterative process. You “fix” one problem, and it is time to examine the next; you expand uptake of HIV care, and you need to do more follow-up; you follow-up and bring more patients back, and you need to expand your adherence support, and so on.

The HIVCore initiative has contributed in many ways to the current HIV implementation agenda as well as sharing of different approaches for research utilization and capacity building for IS. The challenges with the implementation of newer tools and approaches to address the gaps in 90-90-90 coverage are critical for building upon the progress already achieved under the HIVCore initiative.
